# Serum Urate and Incident Cardiovascular Disease: The Coronary Artery Risk Development in Young Adults (CARDIA) Study

**DOI:** 10.1371/journal.pone.0138067

**Published:** 2015-09-18

**Authors:** Huifen Wang, David R. Jacobs, Angelo L. Gaffo, Myron D. Gross, David C. Goff, J. Jeffrey Carr

**Affiliations:** 1 Nutritional Epidemiology Laboratory, Jean Mayer USDA Human Nutrition Research Center on Aging at Tufts University, Boston, MA, United States of America; 2 Division of Epidemiology and Community Health, School of Public Health, University of Minnesota-Twin Cities, Minneapolis, MN, United States of America; 3 Division of Rheumatology, University of Alabama at Birmingham, Birmingham, AL, United States of America; 4 Laboratory of Medicine and Pathology, University of Minnesota-Twin Cities, Minneapolis, MN, United States of America; 5 Colorado School of Public Health, University of Colorado Denver, Aurora, CO, United States of America; 6 Department of Radiology, Vanderbilt University Medical Center, Nashville, TN, United States of America; Mario Negri Institute for Pharmacological Research and Azienda Ospedaliera Ospedali Riuniti di Bergamo, ITALY

## Abstract

**Objective:**

There is controversy about whether serum urate (sUA) predicts future cardiovascular disease (CVD) independently of classical risk factors, and the age at which any prediction starts. We studied the sUA-CVD association among generally healthy adults.

**Methods:**

CARDIA recruited 5115 black and white individuals aged 18–30 years in 1985–1986 (year-0). Fatal and nonfatal CVD events by year 27 (n = 164) were ascertained during annual contacts and classified using medical records. The association with sUA (year-0, 10, 15 and 20) was modeled using Cox proportional hazards regression, pooling over gender-specific quartiles.

**Results:**

Mean sUA concentration was higher in men than women, but increased over time in both genders. Those with elevated sUA had worse metabolic profiles that substantially deteriorated over time. Adjusting for demographic and lifestyle factors (the minimal model), baseline sUA concentration was positively associated with incident CVD (hazard ratio (HR) per mg/dL = 1.21; 95% confidence interval: 1.05, 1.39; P = 0.005). This positive association attenuated to nonsignificance in the full model accounting simultaneously for classical CVD risk factors (HR = 1.09; 0.94, 1.27; P = 0.24). Both the minimal and full models appeared to show stronger associations (than year-0 sUA) between year-10 sUA and incident CVD (HR = 1.27 and 1.12, respectively), but sUA was not statistically significant in the full model. Despite fewer events, year-15 sUA showed a significant sUA-CVD association pattern, with minimal model association magnitude comparable to year-10, and remained significant in the full model (HR = 1.19; 1.02, 1.40; P = 0.03). Hyperuricemia at year-15 strongly predicted CVD risk (HR = 2.11; 1.34, 3.33; P = 0.001), with some attenuation in the full model (HR = 1.68; P = 0.04).

**Conclusions:**

sUA may be an early biomarker for CVD in adults entering middle age. The prediction of CVD by sUA appeared to strengthen with aging. The potential complex relation of sUA with deterioration of a cluster of metabolic abnormalities warrants future exploration.

## Introduction

Urate is the final product of purine catabolism in humans. Elevated serum urate (sUA) can result from excessive dietary intake of purine-rich foods, underexcretion of urate [1,2], or urate overproduction due to enhanced production of xanthine oxidoreductase, an enzyme that catalyzes urate generation [3]. High fructose intake may induce urate generation [4]. Many epidemiological studies have associated elevated sUA concentration with higher risk of cardiovascular disease (CVD) and its risk factors [2]. Meta-analyses [5–7] found marginal to moderate associations of sUA with coronary heart disease (CHD) events, stroke incidence and mortality, and incident heart failure, independent of traditional CVD risk factors. Most existing evidence was in older adults already at higher CVD risk (i.e., greater disorders in classic CVD risk factors, such as body mass index (BMI)) [8],[9]. Elevated sUA concentration appeared to be a circulating marker for classic CVD risk factors in some studies [2], while others inferred a possible causal association between sUA and CVD events [10,11].

In limited evidence concerning younger and healthy populations, a cross-sectional report in 30–45 year olds found that sUA and early atherosclerosis became non-significantly related with adjustment for BMI [12]. We recently reported that repeatedly measured sUA predicted future subclinical atherosclerosis in a cohort of generally healthy people enrolled at ages 18–30 years in the Coronary Artery Risk Development in Young Adults (CARDIA) study [13].We found that sUA through middle age was associated with metabolic factors such as BMI and, that its association with subclinical atherosclerosis when sUA was measured during young adulthood was attenuated by adjustment for BMI. However, the same association with subclinical atherosclerosis when sUA was measured in early middle-age had strengthened, independent of BMI.

In this further longitudinal analysis in CARDIA, we therefore hypothesized that higher sUA concentrations predict incident clinical CVD events. In parallel with our findings for subclinical atherosclerosis [13], we expected that sUA measured in early middle-age (i.e. 4^th^ to 5^th^ decade) in CARDIA would predict CVD events independently of other metabolic factors.

## Methods

### Study sample

CARDIA is a prospective cohort investigating the evolution of CVD risk. Participants were recruited from four field centers in Chicago, IL; Minneapolis, MN; Birmingham, AL; and Oakland, CA [14]. At baseline (1985–1986), 5115 young black and white adults aged 18–30 years were enrolled. Seven follow-up examinations were conducted at years 2, 5, 7, 10, 15, 20 and 25, with response rates among survivors of 91% (*n* = 4624), 86% (*n* = 4352), 81% (*n* = 4086), 79% (*n* = 3950), 74% (*n* = 3672), 72% (*n* = 3549) and 72% (*n* = 3499), respectively. CVD events were monitored through annual contact with all participants (95% successfully contacted at least once between 2009 and 2014). Written and signed informed consent was obtained at every in person visit and also for release of medical records. When there was a separate exam component on a different day, consent was obtained at the initial clinical visit for that examination cycle, with re-explanation on the day of the second contact. There was no consent for interim contacts. The study and procedure for obtaining consent were approved by the institutional review board (IRB) at each field center. This includes the IRBs at the University of Alabama, Birmingham, Northwestern University, University of Minnesota, Kaiser Permanente Division of Research, and National Heart, Lung, and Blood Institute.

### Measurements of serum urate

Participants were asked to fast for at least 12 hours and to avoid smoking and heavy physical activity before the examination. Each participant provided an overnight-fasting blood sample between 7 am and 10 am. Serum samples were shipped (in dry ice) to a central laboratory and stored at -70°C until analysis [15]. sUA concentration was measured at year-0 using the uricase method [16]. A colorimetric assay (modification of the uricase method, in which urate is oxidized to peroxide) [17] was introduced at year-10 and remained in use at years-15 and 20. At year-20, sUA was assessed as part of the Young Adult Longitudinal Trends in Antioxidants (YALTA) ancillary study to CARDIA. In year 17, 105 samples from year 0 were re-assayed using year-15 methods, and a correlation of 0.99 was observed, compared with the original baseline data. Based on re-runs in 2007 of approximately 200 frozen samples per examination and National Institute of Standards Standard Reference Materials, recalibrated values of year-10, 15, and 20 sUA are reported here [13].

### Assessment of any fatal or nonfatal CVD events

Events are ascertained during annual participant contacts; death certificates and relevant medical and hospital records are obtained. The CARDIA Endpoints Surveillance and Adjudication Committee then adjudicates CVD events according to protocol [18]. Incident CVD was defined as adjudicated definite or possible CHD (including myocardial infarction, angina pectoris, and death due to CHD), stroke, transient ischemic attack, heart failure (HF), or peripheral artery disease. Follow-up time was calculated as the days from the sUA measurement date to the day of the earliest incident CVD. No participant at year-0 had a history of clinical CVD events. Results are presented separately for any fatal or nonfatal CVD, CHD (including angina pectoris), HF, and stroke (including transient ischemic attack).

### Measurement of other covariates

The demographic, lifestyle and medical characteristics of participants (e.g., age, race, sex, smoking status and medication use) were assessed during interviews. A validated CARDIA Physical Activity History Questionnaire was administered to measure the exercise activities in the past year [19]. A physical activity score was calculated by multiplying the frequency of participation by the intensity of activity. At the year-0, 7 and 20 examinations, dietary intake was assessed by an interviewer-administered diet history questionnaire specifically developed for CARDIA [20]. Participants were asked to report dietary intake for the past 28 days, including the frequency, amount and preparation methods. The validity and reliability of this diet history questionnaire were evaluated for 12 selected nutrients, and have been described in detail previously [21]. Alcohol consumption was recorded in mL/per week [22]. Daily nutrient intake was calculated using the nutrient database (version 36) developed by the University of Minnesota Nutrition Coordinating Center.

Weight (kg) and height (cm) were measured in clinic following standard protocols; BMI was kg/m^2^. Sitting resting (5 minutes) systolic and diastolic blood pressures were measured three times with a random zero sphygmomanometer through year-15 and subsequently with an Omron model HEM907XL oscillometer (Omron, Mannheim, Germany) calibrated to a parallel random zero measurement. The second and the third measurements were averaged and analyzed.

The details of the laboratory assessment of plasma total cholesterol, HDL cholesterol, triglyceride, fasting (≥8 h) glucose and insulin, and serum creatinine and C-reactive protein (CRP) concentrations have been described previously [13]. Diabetes was defined as fasting glucose ≥126mg/dL or using anti-diabetic medication. Insulin resistance was quantified by using the homeostatic model assessment (HOMA-IR) and calculated as (glucose [mg/dL] × insulin [mU/L])/405 [23]. Metabolic syndrome was defined according to the National Cholesterol Education Program Adult Treatment Panel III [24].

## Statistical Analysis

All analyses were conducted using SAS version 9.3 (SAS Institute, Inc, Cary, NC). All *P*-values were two-sided and statistical significance was set at *P*<0.05. sUA concentration was assessed in 5049, 3870, 3605 and 3147 of the 5115 participants at years-0, 10, 15 and 20, respectively. However, the current study excluded year-20 sUA concentration as an exposure, considering the limited number of CVD events occurring from year 20 to 27. Serial analyses were conducted to predict CVD outcomes using sUA at year-0, year-10, and year-15 in separate models as study exposures (not adjusting for sUA at other exam years simultaneously in the model).

We excluded subjects who had a low glomerular filtration rate (≤60 mL/min per 1.73 m^2^) at any of years-0, 10 or 15 (*n* = 45), leaving a maximum of 5070 participants for any of the following analyses. Compared to the participants who were included, the 45 who were excluded were slightly older and drank more alcohol. The metabolic syndrome prevalence at baseline was also higher among those excluded. For each part of our analysis, anyone with prevalent CVD or missing covariate data at the year of sUA measurement was further excluded from the 5070 participants. Therefore, the sample size varied across sUA exposures and covariates at different exam years, as reported for each specific analysis in the tables.

The concentration of sUA was modeled in three ways: (1) as a continuous variable; (2) as gender-specific tertiles that were pooled to incorporate the potential gender difference in sUA concentrations [25]; (3) as hyperuricaemia (i.e. sUA ≥6.8 mg/dL [26,27]), the concentration above which sUA becomes insoluble and starts depositing as monosodium urate in tissues, which is the precursor of gout. Participant characteristics (i.e. in means or proportions) were described by gender-specific sUA tertiles.

Multiple Cox proportional hazards regression models were used to examine the longitudinal association between sUA and CVD. For each regression model, covariates including sex, race, age, clinic, education, total energy, alcohol, and protein intakes at the year of sUA measurement were adjusted. Additionally, we adjusted for lifestyle factors (e.g. smoking status), BMI, metabolic syndrome (yes vs. no), insulin resistance, the ratio of total cholesterol and HDL-cholesterol, blood pressure, and waist circumference as the classical CVD risk factors, plus the usage of anti-hypertension medication, the usage of diuretics, and glomerular filtration rate (as a continuous variable).

No significant sex- or race-interaction was found. Because both sUA concentrations [25] and CVD risk [28] may differ between men and women, we also report a sensitivity analysis stratified by gender.

## Results

### Participant characteristics

Mean sUA concentrations increased over time in both genders. The mean (SD) sUA concentrations at years-0, 10 and 15 in men were 6.16 (1.15), 6.35 (1.24), and 6.42 (1.27) mg/dL, respectively; and in women were 4.46 (0.97), 4.63 (1.00), and 4.75 (1.07) mg/dL, respectively. Metabolic profiles substantially deteriorated over time (**Table 1**).

**Table 1:** Unadjusted participant characteristics (mean±SD or percent) of participants across exam years.

	Year 0	Year 10	Year 15
N ^a^	4816	3731	3492
Age (year)	24.9±3.6	35.0±3.7	40.2±3.6
Women (%)	54.8	55.3	55.6
Whites (%)	49.5	52.4	53.8
Former smoker (%)	13.5	16.5	18.3
Current smoker (%)	29.7	25.0	21.5
BMI (kg/m^2^)	24.5±5.0	27.5±6.5	28.8±6.8
Waist circumference (cm)	77.7±11.3	85.9±14.4	89.5±15.0
Metabolic syndrome (%)	2.3	10.6	17.2
Systolic blood pressure (mmHg)	110.3±10.8	109.8±12.6	113.1±14.8
Diastolic blood pressure (mmHg)	68.5±9.6	72.3±10.1	74.4±11.5
Glomerular filtration rate (mL/min per 1.73 m^2^)	124.1±15.5	110.8±23.0	104.6±16.1
Use anti-hypertension medication (%)	2.1	3.0	6.9
Use diuretics (%)	1.0	1.3	3.5
Biochemical markers			
Total cholesterol (mg/dL)	176.7±33.4	178.0±34.4	184.9±35.7
HDL-C (mg/dL)	53.2±13.2	50.2±14.0	50.8±14.6
LDL-C (mg/dL)	109.1±31.2	109.3±32.0	113.2±32.2
Triglycerides (mg/dL)	72.4±46.3	91.4±72.3	105.1±93.1
Creatinine (mg/dL)	0.80±0.13	0.83±0.17	0.85±0.18
Glucose (mg/dL)	82.3±13.9	91.3±17.0	94.0±20.1
Insulin (μU/mL)	7.6±5.6	9.7±6.8	10.2±7.7
HOMA-IR ^b^	1.6±1.4	2.3±2.2	2.5±2.4

^a^ Prevalent CVD endpoints and missing data on sUA or covariates at the year of sUA measurement were excluded from the analyses.

^b^ HOMA-IR was calculated as (glucose × insulin) / 405 [23].

### Association of sUA concentration with incident CVD

Over the 27-year follow-up, 164, 79, 40, and 40 participants developed any fatal or non-fatal CVD, CHD, CHF or stroke, respectively. Adjusting for baseline age, sex, race, clinic, education, physical activity, smoking and intakes of alcohol, protein and total energy (**Table 2**), baseline sUA concentration was positively associated with the incident CVD by year 27 (hazard ratio (HR) per mg/dL of sUA = 1.21; 95% confidence interval (CI) = 1.05, 1.39). Compared to participants with sUA concentration in the lowest tertile at year-0, those in the highest tertile had a greater risk (HR = 1.58; 95%CI = 1.08, 2.31) for any fatal or non-fatal CVD by year 27. This positive association was substantially attenuated with further adjustment for baseline BMI, systolic and diastolic blood pressure, anti-hypertension medication use (excluding those taking diuretics), diuretics use, and glomerular filtration rate (HR = 1.09, P = 0.24). Hyperuricemia at baseline related positively to incident CVD by year 27, with findings attenuated by adjustment.

**Table 2:** Longitudinal association between sUA and the incidence of any fatal or nonfatal CVD endpoints by year 27.

	Tertiles of sUA concentrations						
	Q1	Q2	Q3	HR per mg/dL sUA^h^	*P* ^i^	Hyperuricemia	*P*
*Y0 sUA* (*n* = 4816, 164 CVD events)							
Y0 sUA concentration in men (median and range)	5.10 (1.10, 5.60)	6.10 (5.70, 6.50)	7.20 (6.60, 11.20)				
Y0 sUA concentration in women (median and range)	3.60 (1.00, 4.00)	4.40 (4.10, 4.70)	5.40 (4.80, 8.80)				
No. of people at risk	1674	1485	1657			637 (4179) ^j^	
No. of CVD cases	48	49	67			36 (128) ^j^	
CVD rates/1000 person-years	1.147	1.321	1.621			2.302 (1.223) ^j^	
Model 1 ^a^	1.00	1.28 (0.86, 1.92) ^g^	1.58 (1.08, 2.31)	1.21 (1.05, 1.39)	0.005	1.71 (1.13, 2.58) ^k^	0.01
Full multivariable Model 1 ^b^	1.00	1.10 (0.73, 1.66)	1.22 (0.81, 1.84)	1.09 (0.94, 1.27)	0.24	1.40 (0.92, 2.15)	0.12
*Y10 sUA* (*n* = 3731, 133 CVD events)							
Y10 sUA concentration in men (median and range)	5.23 (3.01, 5.74)	6.24 (5.84, 6.75)	7.56 (6.85, 12.11)				
Y0 sUA concentration in women (median and range)	3.71 (2.20, 4.12)	4.52 (4.22, 4.93)	5.54 (5.03, 9.89)				
No. of people at risk	1286	1232	1213			600 (3131)	
No. of CVD cases	30	41	62			35 (98)	
CVD rates/1000 person-years	1.498	2.140	3.331			3.821 (2.015)	
Model 2 ^c^	1.00	1.59 (0.98, 2.56)	2.18 (1.40, 3.40)	1.27 (1.11, 1.46)	<0.001	1.46 (0.95, 2.24)	0.08
Full multivariable Model 2 ^d^	1.00	1.37 (0.84, 2.21)	1.55 (0.95, 2.51)	1.12 (0.96, 1.31)	0.14	1.08 (0.68, 1.69)	0.75
*Y15 sUA* (*n* = 3492, 98 CVD events)							
Y15 sUA concentration in men (median and range)	5.15 (3.34, 5.72)	6.29 (5.82, 6.77)	7.62 (6.86, 11.91)				
Y15 sUA concentration in women (median and range)	3.72 (2.10, 4.10)	4.58 (4.20, 5.05)	5.72 (5.15, 11.05)				
No. of people at risk	1104	1225	1163			603 (2889)	
No. of CVD cases	23	25	50			35 (63)	
CVD rates/1000 person-years	1.954	1.913	4.086			5.554 (2.047)	
Model 3 ^e^	1.00	0.92 (0.52, 1.62)	1.71 (1.04, 2.82)	1.27 (1.11, 1.46)	0.001	2.11 (1.34, 3.33)	0.001
Full multivariable Model 3 ^f^	1.00	0.85 (0.47, 1.51)	1.34 (0.78, 2.31)	1.19 (1.02, 1.40)	0.03	1.68 (1.04, 2.71)	0.04

sUA, serum urate; CVD, cardiovascular disease; Y, year; Q, quartile; BMI, body mass index; CI, confidence interval.

^a^ Model 1: adjusted for year 0 age, sex, race, clinic, education level, smoking status, physical activity and intakes of total calories, alcohol and protein.

^b^ Model 1 + year 0 BMI, systolic and diastolic blood pressure, anti-hypertension medication use (excluding those taking diuretics), diuretics use, and glomerular filtration rate.

^c^ Model 2: adjusted for age, sex, race, clinic, education level, smoking status and physical activity at year 10, and average intakes of total calories, alcohol and protein at years 0 and 7.

^d^ Model 2 + year 10 BMI, systolic and diastolic blood pressure, anti-hypertension medication use (excluding those taking diuretics), diuretics use, and glomerular filtration rate.

^e^ Model 3: adjusted for age, sex, race, clinic, education level, smoking status and physical activity at year 15, and average intakes of total calories, alcohol and protein at years 0 and 7.

^f^ Model 3 + year 15 BMI, systolic and diastolic blood pressure, anti-hypertension medication use (excluding those taking diuretics), diuretics use, and glomerular filtration rate

^g^ Hazard ratio (95% CI) for the incidence of any fatal or nonfatal CVD endpoints by the end of 2012 (year 25) across sUA tertiles, reference group is participants in the lowest tertile of sUA concentrations.

^h^ Hazard ratio (95% CI) for the incidence of any fatal or nonfatal CVD endpoints per mg/dL sUA when using continuous sUA variable.

^i^ *P*-values for the association between sUA and CVD when using continuous sUA variables.

^j^ Values are presented as “hyperuricemia group (reference group)”. Numbers in the reference group (participants without hyperuricaemia, i.e. sUA <6.8 mg/dL) are given in parentheses.

^k^Hazard ratio (95% CI) for the incidence of any fatal or nonfatal CVD endpoints by the end of 2012 (year 25) for the hyperuricemia group.

sUA at both year-10 and year-15 had larger estimated magnitude of association with incident CVD by year 27 (both HR = 1.27, P≤0.001) than was found for sUA at year-0. Prediction from sUA at year-10 (based on 133 incident CVD events) was attenuated in the full multivariable model, but remained significant with additional adjustment for individual year-10 BMI, metabolic syndrome, total cholesterol/HDL-C, HOMA-IR, or blood pressure (data not shown). The association between sUA at year-15 and incident CVD, adjusting for demographic and lifestyle factors, was of a comparable magnitude to that at year-10; and remained significant but moderate in the full model (HR = 1.19; 1.02, 1.40; P = 0.03). Being hyperuricemic at year-15 significantly predicted CVD development, even after adjustment for metabolic factors.

In sensitivity analysis, the estimated association between sUA and CVD was nominally stronger in men than in women (**S1 Table**); p<0.05 for sex-interaction with sUA only at year-15. Like associations with incident CVD, associations with incident CHD, HF, and stroke with sUA concentrations (each examined separately) were generally positive and estimates strengthened when predicting from at years-10 and 15 vs. prediction from year-0 (**S2, S3 and S4 Tables**). However, associations for separate outcome were not statistically significant.

## Discussion

In this young adult cohort that was generally healthy at baseline, a higher sUA concentration was associated with a moderate and significant risk of developing any fatal or non-fatal CVD over a 27-year follow-up. The prediction of CVD based on sUA concentration in young adults was largely attributable to the correlation of sUA with classical CVD risk factors, but appeared to strengthen for sUA measured in early middle age. The sUA concentrations were highly correlated across examinations and those with elevated sUA concentrations had worse metabolic profiles, as previously reported [13]. Coupled with the deterioration in multiple classic CVD risk factors with aging, sUA appeared to be involved as a part of a complex web of changing metabolic variables over early adult life in its prediction of CVD endpoints.

Our present findings were generally consistent with our report on the positive association between sUA concentration and the risk of subclinical atherosclerosis (comprising coronary artery calcified plaque and maximum common carotid intima-media thickness) [13]. In both studies, we found that the associations of sUA with subclinical atherosclerosis and CVD were more substantial in men than in women, although the sex-interaction was generally not statistically significant in the present analyses. The prediction of both subclinical atherosclerosis and CVD endpoints strengthened using sUA concentration in early middle age compared to sUA in young adulthood. Of note, some previous studies reported that the association between sUA and CVD outcomes (or intermediate outcomes) was stronger in women than in men [5], while others showed the opposite [29–32]. While this potential gender interaction is interesting, it is not clearly significant in our study and deserves further study.

Higher sUA concentration usually clustered with several other metabolic abnormalities such as high blood pressure, hyperinsulinemia, and dyslipidemia [2,33]; sUA also predicted future components of metabolic syndrome [34]. The role of elevated sUA concentration in CVD development has long been debated, particularly whether its predictive capacity added value to the classic CVD risk factors. A few large prospective cohort studies concluded that there was no independent association of sUA with CHD [35,36]; whereas, others found significant association between sUA and CVD with full adjustment for classic CVD risk factors [11,37]. Using Mendelian Randomization, some studies failed to find any evidence showing a causal relationship between sUA and CVD [38,39], while one study in men of average age 70 years and followed for a median of 10 years found that a genetic risk that correlated with sUA was associated with cardiovascular and sudden cardiac death [40]. A few meta-analyses of older adults have reported an attenuated, but significant small to modest association between sUA and CVD outcomes after adjustment for traditional CVD risk factors [5–7]. Older adults generally have metabolic profiles that are deteriorated compared to our CARDIA sample. Therefore, the independent role of sUA found in older adults supports our observation in CARDIA that sUA significantly predicts CVD outcomes independent of traditional CVD risk factor profiles, starting in early middle age. No previous studies have examined how the prediction of CVD from sUA progressed as the sample aged.

Knowles and Reaven recently suggested that sUA *per se* was a biomarker of insulin resistance in CVD development [41]. Nevertheless, we found that adjusting for insulin resistance or other individual metabolic abnormalities did not completely explain the significant positive association of elevated sUA measured in early middle age with incident clinical CVD risk. Although measurement error in insulin resistance cannot be excluded, as we previously summarized [13], several mechanisms may be involved in the sUA-CVD association, including elevated sUA (1) being a marker of xanthine oxidoreductase activity and oxidative stress related to atherosclerosis; (2) mediating proinflammatory pathways; (3) inducing endothelial dysfunction; (4) activating the renin–angiotensin system; (5) resulting from renal dysfunction leading to reduced sUA clearance (also suggested by Knowles and Reaven [41]). Gaffo et al. found in CARDIA that higher sUA, even for sUA<6.8mg/dL, independently predicted subsequent incident hypertension [26]. Given similar findings elsewhere [42], a 2-stage urate-mediated hypertension mechanism was proposed [43]. It is suspected that increased sUA activates the renin-angiotensin system and reduces the bioavailability of circulating plasma nitrates, resulting in elevated blood pressure. During this first stage, the metabolic deterioration may still be reversible by decreasing sUA concentration. In the second stage, vascular wall thickening and smooth muscle proliferation is thought to occur due to increased oxidative stress; the resulting hypertension would be less responsive to serum urate lowering and lead to adverse cardiovascular outcomes [42,43]. This hypothesis is supported by our current findings because sUA-CVD association in early adulthood was eliminated after adjustment for blood pressure, but it remained significant in middle-age. Thus, we suspect that elevated sUA concentration shares much but not all common pathophysiology with each individual metabolic disorder in CVD development. Although we cannot determine whether reducing sUA concentration would yield clinical benefit, it might be important to track sUA concentration in clinical practice and to conduct further research to better understand sUA-related pathways. In other words, sUA influence in CVD development in early adulthood might still not be evident; whereas as people age, sUA rises, and vascular remodeling is established, the impact of sUA or its precursors may become more evident.

The positive associations of sUA with CHD, stroke, and HF events generally did not achieve statistical significance, consistent with fewer events of these separate outcomes. sUA may have different pathophysiologies between separate CVD outcomes. In a recent systematic review, elevated sUA was related to increased risk of incident HF as well as CVD and all-cause mortality in HF patients; the majority of studies involved middle-aged and elderly adults and the authors cautioned that the culprit for HF development might be unregulated xanthine oxidase rather than sUA itself [7].

Study limitations include that generalizations to older populations and drawing causal inferences should be cautious. Because this cohort has just entered late middle-age and our analyses were based on only 164 incident CVD cases, our statistical power was limited, especially in the analyses using year 15 sUA as an exposure, in examining CVD subtypes (i.e. CHD, stroke and HF), and in studying potential gender-interaction.

Study strengths include that we followed young, healthy adults for 27 years from young adulthood to middle-age, while most of the previous evidence focused on older adults. The current longitudinal design with repeated measurements of both exposures and outcomes provided new evidence to the debate on sUA and future CVD. We carefully tested the potential impacts of classic CVD risk factors, either individually or as a cluster, on the sUA and CVD association.

In conclusion, we observed a significantly positive correlation between sUA concentration and the development of CVD outcomes among young to middle-aged adults during a 27-year follow-up. CVD event prediction based on sUA appeared to strengthen with age, but the magnitude of the prediction was only small to moderate. During young adulthood, sUA may be responsive to individual metabolic abnormalities, while by middle-age adjusting for individual metabolic disorders did not seem to fully diminish the sUA and CVD association. Our study suggested that monitoring sUA concentrations may be of clinical importance to CVD prevention and management. Because the CARDIA study is still on-going and we expect to obtain additional data on this adult cohort as it ages, it would be interesting to extend our analysis with future follow-up to see whether the role of sUA as a predictor continues to evolve as the cohort ages.

## Supporting Information

S1 Table Longitudinal association between sUA and the incidence of any fatal or nonfatal CHD by year 27. (DOCX)

S2 Table Longitudinal association between sUA and the incidence of any CHF endpoints by year 27. (DOCX)

S3 Table Longitudinal association between sUA and the incidence of any stroke (fatal and non-fatal) endpoints by year 27. (DOCX)

S4 Table Longitudinal association between sUA and the incidence of any fatal or nonfatal CVD endpoints by year 27, stratified by sex. (DOCX)
