# Supplementary material for: Serum Urate and Incident Cardiovascular Disease: The Coronary Artery Risk Development in Young Adults (CARDIA) Study
Source: PLoS One. 2015 Sep 18;10(9):e0138067. doi: 10.1371/journal.pone.0138067 (PMC4575092; doi:10.1371/journal.pone.0138067)
Supplement: S2 Table — (DOCX) [file pone.0138067.s002.docx]

**Supporting Information Table S2.** Longitudinal association between sUA and the incidence of any CHF endpoints by year 27

|  | Tertiles of sUA concentrations | | |  |  |  |  |
| --- | --- | --- | --- | --- | --- | --- | --- |
|  | Q1 | Q2 | Q3 | HR per mg/dL sUA^h^ | *P* ^i^ | Hyperuricemia | *P* |
| *Y0 sUA* (*n* = 4815, 40 CHF events) | | |  |  |  |  |  |
| Y0 sUA concentration in men (median and range) | 5.10 (1.10, 5.60) | 6.10 (5.70, 6.50) | 7.20 (6.60, 11.20) |  |  |  |  |
| Y0 sUA concentration in women (median and range) | 3.60 (1.00, 4.00) | 4.40 (4.10, 4.70) | 5.40 (4.80, 8.80) |  |  | 637 (4179) ^j^ |  |
| No. of CHF cases | 13 | 10 | 17 |  |  | 10 (30) |  |
| CHF rates/1000 person-years | 0.309 | 0.268 | 0.409 |  |  | 0.634 (0.285) |  |
| Model 1 ^a^ | 1.00 | 1.03 (0.45, 2.37) ^g^ | 1.55 (0.74, 3.24) | 1.27 (0.97, 1.67) | 0.08 | 2.32 (1.03, 5.19) ^k^ | 0.04 |
| Full Multivariate Model 1 ^b^ | 1.00 | 0.84 (0.36, 1.95) | 1.03 (0.46, 2.31) | 1.09 (0.81, 1.46) | 0.56 | 1.79 (0.78, 4.09) | 0.17 |
|  |  |  |  |  |  |  |  |
| *Y10 sUA* (*n* = 3730, 33 CHF events) | | |  |  |  |  |  |
| Y10 sUA concentration in men (median and range) | 5.23 (3.01, 5.74) | 6.24 (5.84, 6.75) | 7.56 (6.85, 12.11) |  |  |  |  |
| Y10 sUA concentration in women (median and range) | 3.71 (2.20, 4.12) | 4.52 (4.22, 4.93) | 5.54 (5.03, 9.89) |  |  |  |  |
| No. of people at risk | 1286 | 1232 | 1213 |  |  | 600 (3131) |  |
| No. of CHF cases | 7 | 12 | 14 |  |  | 9 (24) |  |
| CHF rates/1000 person-years | 0.348 | 0.621 | 0.743 |  |  | 0.970 (0.490) |  |
| Model 2 ^c^ | 1.00 | 1.95 (0.77, 4.99) | 1.91 (0.76, 4.78) | 1.28 (1, 1.66) | 0.06 | 1.30 (0.56, 3.03) | 0.54 |
| Full Multivariate Model 2 ^d^ | 1.00 | 1.40 (0.54, 3.64) | 0.90 (0.33, 2.46) | 0.99 (0.72, 1.36) | 0.96 | 0.74 (0.30, 1.82) | 0.51 |
|  |  |  |  |  |  |  |  |
| *Y15 sUA* (*n* = 3491, 23 CHF events) | | |  |  |  |  |  |
| Y15 sUA concentration in men (median and range) | 5.15 (3.34, 5.72) | 6.29 (5.82, 6.77) | 7.62 (6.86, 11.91) |  |  |  |  |
| Y15 sUA concentration in women (median and range) | 3.72 (2.10, 4.10) | 4.58 (4.20, 5.05) | 5.72 (5.15, 11.05) |  |  |  |  |
| No. of people at risk | 1104 | 1225 | 1163 |  |  | 603 (2889) |  |
| No. of CHF cases | 6 | 6 | 11 |  |  | 7 (16) |  |
| CHF rates/1000 person-years | 0.507 | 0.457 | 0.888 |  |  | 1.091 (0.517) |  |
| Model 3 ^e^ | 1.00 | 0.94 (0.30, 2.94) | 1.46 (0.53, 4.01) | 1.22 (0.91, 1.64) | 0.17 | 1.55 (0.59, 4.08) | 0.38 |
| Full Multivariate Model 3 ^f^ | 1.00 | 0.77 (0.23, 2.62) | 0.93 (0.29, 2.91) | 1.09 (0.78, 1.54) | 0.60 | 1.05 (0.38, 2.92) | 0.93 |

sUA, serum urate; CHF, congestive heart failure; Y, year; Q, quartile; BMI, body mass index; CI, confidence interval.

^a^ Model 1: adjusted for year 0 age, sex, race, clinic, education level, smoking status, physical activity and intakes of total calories, alcohol and protein.

^b^ Model 1 + year 0 BMI, systolic and diastolic blood pressure, anti-hypertension medication use (excluding those taking diuretics), diuretics use, and glomerular filtration rate.

^c^ Model 2: adjusted for age, sex, race, clinic, education level, smoking status and physical activity at year 10, and average intakes of total calories, alcohol and protein at years 0 and 7.

^d^ Model 2 + year 10 BMI, systolic and diastolic blood pressure, anti-hypertension medication use (excluding those taking diuretics), diuretics use, and glomerular filtration rate.

^e^ Model 3: adjusted for age, sex, race, clinic, education level, smoking status and physical activity at year 15, and average intakes of total calories, alcohol and protein at years 0 and 7.

^f^ Model 3 + year 15 BMI, systolic and diastolic blood pressure, anti-hypertension medication use (excluding those taking diuretics), diuretics use, and glomerular filtration rate

^g^ Hazard ratio (95% CI) for the incidence of any CHF endpoints by the end of 2012 (year 25) across sUA tertiles, reference group is participants in the lowest tertile of sUA concentrations.

^h^ Hazard ratio (95% CI) for the incidence of any CHD endpoints per mg/dL sUA when using continuous sUA variable.

^i^ *P*-values for the association between sUA and CHF when using continuous sUA variables.

^j^ Values are presented as “hyperuricemia group (reference group)”. Reference group is participants without hyperuricaemia (i.e. sUA <6.8 mg/dL).

^k^ Hazard ratio (95% CI) for the incidence of any CHF endpoints by the end of 2012 (year 25) for the hyperuricemia group.
