# Supplementary material for: Serum Urate and Incident Cardiovascular Disease: The Coronary Artery Risk Development in Young Adults (CARDIA) Study
Source: PLoS One. 2015 Sep 18;10(9):e0138067. doi: 10.1371/journal.pone.0138067 (PMC4575092; doi:10.1371/journal.pone.0138067)
Supplement: S3 Table — (DOCX) [file pone.0138067.s003.docx]

**Supporting Information Table S3.** Longitudinal association between sUA and the incidence of any stroke (fatal and non-fatal) endpoints by year 27

|  | Tertiles of sUA concentrations | | |  |  |  |  |
| --- | --- | --- | --- | --- | --- | --- | --- |
|  | Q1 | Q2 | Q3 | HR per mg/dL sUA ^h^ | *P* ^i^ | Hyperuricemia | *P* |
| *Y0 sUA* (*n* = 4815, 40 stroke events) | | |  |  |  |  |  |
| Y0 sUA concentration in men (median and range) | 5.10 (1.10, 5.60) | 6.10 (5.70, 6.50) | 7.20 (6.60, 11.20) |  |  |  |  |
| Y0 sUA concentration in women (median and range) | 3.60 (1.00, 4.00) | 4.40 (4.10, 4.70) | 5.40 (4.80, 8.80) |  |  | 637 (4179) ^j^ |  |
| No. of stroke cases | 16 | 8 | 23 |  |  | 6 (41) |  |
| stroke rates/1000 person-years | 0.381 | 0.214 | 0.553 |  |  | 0.380 (0.390) |  |
| Model 1 ^a^ | 1.00 | 0.70 (0.28, 1.58) ^g^ | 1.77 (0.92, 3.43) | 1.14 (0.88, 1.47) | 0.31 | 1.27 (0.49, 3.26) ^k^ | 0.62 |
| Full Multivariate Model 1 ^b^ | 1.00 | 0.67 (0.28, 1.60) | 1.83 (0.88, 3.78) | 1.11 (0.83, 1.49) | 0.48 | 1.17 (0.44, 3.11) | 0.76 |
|  |  |  |  |  |  |  |  |
| *Y10 sUA* (*n* = 3730, 36 stroke events) | | |  |  |  |  |  |
| Y10 sUA concentration in men (median and range) | 5.23 (3.01, 5.74) | 6.24 (5.84, 6.75) | 7.56 (6.85, 12.11) |  |  |  |  |
| Y10 sUA concentration in women (median and range) | 3.71 (2.20, 4.12) | 4.52 (4.22, 4.93) | 5.54 (5.03, 9.89) |  |  |  |  |
| No. of people at risk | 1286 | 1232 | 1213 |  |  | 600 (3131) |  |
| No. of stroke cases | 9 | 12 | 20 |  |  | 5 (36) |  |
| stroke rates/1000 person-years | 0.447 | 0.621 | 1.061 |  |  | 0.536 (0.735) |  |
| Model 2 ^c^ | 1.00 | 1.61 (0.66, 3.96) | 2.35 (1.03, 5.38) | 1.3 (1.01, 1.67) | 0.04 | 0.76 (0.27, 2.12) | 0.60 |
| Full Multivariate Model 2 ^d^ | 1.00 | 1.44 (0.57, 3.60) | 1.76 (0.71, 4.34) | 1.20 (0.89, 1.61) | 0.23 | 0.59 (0.20, 1.73) | 0.33 |
|  |  |  |  |  |  |  |  |
| *Y15 sUA* (*n* = 3491, 27 stroke events) | | |  |  |  |  |  |
| Y15 sUA concentration in men (median and range) | 5.15 (3.34, 5.72) | 6.29 (5.82, 6.77) | 7.62 (6.86, 11.91) |  |  |  |  |
| Y15 sUA concentration in women (median and range) | 3.72 (2.10, 4.10) | 4.58 (4.20, 5.05) | 5.72 (5.15, 11.05) |  |  |  |  |
| No. of people at risk | 1104 | 1225 | 1163 |  |  | 603 (2889) |  |
| No. of stroke cases | 6 | 4 | 17 |  |  | 9 (22) |  |
| stroke rates/1000 person-years | 0.507 | 0.457 | 1.537 |  |  | 1.405 (0.711) |  |
| Model 3 ^e^ | 1.00 | 0.78 (0.25, 2.44) | 2.22 (0.88, 5.60) | 1.34 (1.04, 1.72) | 0.03 | 2.22 (0.92, 5.35) | 0.07 |
| Full Multivariate Model 3 ^f^ | 1.00 | 0.83 (0.26, 2.63) | 2.12 (0.76, 5.93) | 1.33 (0.99, 1.80) | 0.06 | 1.94 (0.73, 5.17) | 0.19 |

sUA, serum urate; Y, year; Q, quartile; BMI, body mass index; CI, confidence interval.

^a^ Model 1: adjusted for year 0 age, sex, race, clinic, education level, smoking status, physical activity and intakes of total calories, alcohol and protein.

^b^ Model 1 + year 0 BMI, systolic and diastolic blood pressure, anti-hypertension medication use (excluding those taking diuretics), diuretics use, and glomerular filtration rate.

^c^ Model 2: adjusted for age, sex, race, clinic, education level, smoking status and physical activity at year 10, and average intakes of total calories, alcohol and protein at years 0 and 7.

^d^ Model 2 + year 10 BMI, systolic and diastolic blood pressure, anti-hypertension medication use (excluding those taking diuretics), diuretics use, and glomerular filtration rate.

^e^ Model 3: adjusted for age, sex, race, clinic, education level, smoking status and physical activity at year 15, and average intakes of total calories, alcohol and protein at years 0 and 7.

^f^ Model 3 + year 15 BMI, systolic and diastolic blood pressure, anti-hypertension medication use (excluding those taking diuretics), diuretics use, and glomerular filtration rate

^g^ Hazard ratio (95% CI) for the incidence of any fatal or nonfatal stroke endpoints by the end of 2012 (year 25) across sUA tertiles, reference group is participants in the lowest tertile of sUA concentrations.

^h^ Hazard ratio (95% CI) for the incidence of any fatal or nonfatal stroke endpoints per mg/dL sUA when using continuous sUA variable.

^i^ *P*-values for the association between sUA and stroke when using continuous sUA variables.

^j^ Values are presented as “hyperuricemia group (reference group)”. Reference group is participants without hyperuricaemia (i.e. sUA <6.8 mg/dL).

^k^ Hazard ratio (95% CI) for the incidence of any fatal or nonfatal stroke endpoints by the end of 2012 (year 25) for the hyperuricemia group.
