# Supplementary material for: Serum Urate and Incident Cardiovascular Disease: The Coronary Artery Risk Development in Young Adults (CARDIA) Study
Source: PLoS One. 2015 Sep 18;10(9):e0138067. doi: 10.1371/journal.pone.0138067 (PMC4575092; doi:10.1371/journal.pone.0138067)
Supplement: S4 Table — (DOCX) [file pone.0138067.s004.docx]

**Supporting Information Table S4:** Longitudinal association between sUA and the incidence of any fatal or nonfatal CVD endpoints by year 27, stratified by sex

|  | *Men* | | *Women* | | |
| --- | --- | --- | --- | --- | --- |
|  | HR per mg/dL sUA^g^ | *P* ^h^ | HR per mg/dL sUA | *P* |  |
| *Y0 sUA* (*n* =4816) | | |  |  |  |
| Y0 sUA concentration (median and range) | 6.10 (1.10, 11.20) |  | 4.40 (1.00, 8.80) |  |  |
| No. of people at risk | 2177 |  | 2639 |  |  |
| No. of CVD cases | 90 |  | 74 |  |  |
| CVD rates/1000 person-years | 1.678 |  | 1.110 |  |  |
| Model 1 ^a^ | 1.27 (1.07, 1.52) | 0.007 | 1.16 (0.94, 1.44) | 0.18 |  |
| Full multivariable Model 1 ^b^ | 1.16 (0.96, 1.41) | 0.13 | 1.01 (0.79, 1.29) | 0.96 |  |
|  |  |  |  |  |  |
| *Y10 sUA* (*n* = 3730) | | |  |  |  |
| Y10 sUA concentration (median and range) | 6.24 (3.01, 12.11) |  | 4.52 (2.20, 9.89) |  |  |
| No. of people at risk | 1667 |  | 2064 |  |  |
| No. of CVD cases | 77 |  | 56 |  |  |
| CVD rates/1000 person-years | 2.997 |  | 1.745 |  |  |
| Model 2 ^c^ | 1.35 (1.13, 1.61) | 0.001 | 1.22 (0.98, 1.52) | 0.08 |  |
| Full multivariable Model 2 ^d^ | 1.21 (0.99, 1.47) | 0.06 | 1.03 (0.77, 1.36) | 0.86 |  |
|  |  |  |  |  |  |
| *Y15 sUA* (*n* = 3491) | | |  |  |  |
| Men |  |  |  |  |  |
| Y15 sUA concentration (median and range) | 6.29 (3.34, 11.91) |  | 4.58 (2.10, 11.05) |  |  |
| No. of people at risk | 1550 |  | 1942 |  |  |
| No. of CVD cases | 56 |  | 42 |  |  |
| CVD rates/1000 person-years | 3.409 |  | 2.034 |  |  |
| Model 3 ^e^ | 1.46 (1.23, 1.74) | <0.001 | 1 (0.76, 1.32) | 0.98 |  |
| Full multivariable Model 3 ^f^ | 1.44 (1.18, 1.76) | <0.001 | 0.81 (0.57, 1.13) | 0.21 |  |

sUA, serum urate; CAC, coronary artery calcified plaque; Y, year; Q, quartile; BMI, body mass index; CI, confidence interval.

^a^ Model 1: adjusted for year 0 age, race, clinic, education level, smoking status, physical activity and intakes of total calories, alcohol and protein. *P*-value for testing sUA-sex interaction=0.39 in this model.

^b^ Model 1 + year 0 BMI, systolic and diastolic blood pressure, anti-hypertension medication use (excluding those taking diuretics), diuretics use, and glomerular filtration rate.

^c^ Model 2: adjusted for age, race, clinic, education level, smoking status and physical activity at year 10, and average intakes of total calories, alcohol and protein at years 0 and 7. *P*-value for testing sUA-sex interaction=0.66 in this model.

^d^ Model 2 + year 10 BMI, systolic and diastolic blood pressure, anti-hypertension medication use (excluding those taking diuretics), diuretics use, and glomerular filtration rate.

^e^ Model 3: adjusted for age, race, clinic, education level, smoking status and physical activity at year 15, and average intakes of total calories, alcohol and protein at years 0 and 7. *P*-value for testing sUA-sex interaction=0.03 in this model.

^f^ Model 3 + year 15 BMI, systolic and diastolic blood pressure, anti-hypertension medication use (excluding those taking diuretics), diuretics use, and glomerular filtration rate

^g^ Hazard ratio (95% CI) for incidence of any fatal or nonfatal CVD endpoints per mg/dL sUA when using continuous sUA variable.

^h^ *P*-values for the association between sUA and CVD when using continuous sUA variables.
